# Early Assessment of Colorectal Cancer Patients with Liver Metastases Treated with Antiangiogenic Drugs: The Role of Intravoxel Incoherent Motion in Diffusion-Weighted Imaging

**DOI:** 10.1371/journal.pone.0142876

**Published:** 2015-11-13

**Authors:** Vincenza Granata, Roberta Fusco, Orlando Catalano, Salvatore Filice, Daniela Maria Amato, Guglielmo Nasti, Antonio Avallone, Francesco Izzo, Antonella Petrillo

**Affiliations:** 1 Department of Radiology, “Istituto Nazionale Tumori IRCCS Fondazione Pascale, via Mariano Semmola, Naples, I-80131, Italy; 2 Department of Abdominal Oncology, “Istituto Nazionale Tumori IRCCS Fondazione Pascale, via Mariano Semmola, Naples, I-80131, Italy; 3 Department of Hepatobiliary Surgical Oncology, “Istituto Nazionale Tumori IRCCS Fondazione Pascale, via Mariano Semmola, Naples, I-80131, Italy; Northwestern University Feinberg School of Medicine, UNITED STATES

## Abstract

**Purpose:**

To assess the feasibility and effectiveness of quantitative intravoxel incoherent motion (IVIM) of Diffusion-weighted imaging (DWI) in the assessment of liver metastases treated with targeted chemotherapy agents.

**Methods:**

12 patients with unresectable liver metastases from colorectal cancer were enrolled and received neoadjuvant FOLFIRI (5-fluorouracil, leucovorin, irinotecan) plus bevacizumab therapy. DWI was performed for 36 metastases at baseline and after 14 days from starting the treatment. In addition to the basic IVIM metrics, the product between pseudo-diffusivity and perfusion fraction was considered as a descriptor roughly analogous to the flow. Median diffusion parameters of Region of Interest (ROI) were used as representative values for each lesion. Normalized parameters in comparison with the median value of spleen were also collected. The percentual change of the diffusion parameters was calculated. The response to chemotherapy was evaluated according the Response Evaluation Criteria in Solid Tumors (RECIST) as calculated on whole-body CT scan obtained three months after treatment. Mann Whitney test and Receiver operating characteristic (ROC) analysis were performed.

**Results:**

24 lesions were categorized as responding and 12 as not responding. There was no statistically significant difference among absolute and normalized diffusion parameters between the pretreatment and the post-treatment findings. Instead, the perfusion fraction (f_p_) values showed a statistical difference between responder and non-responder lesions: sensitivity and specificity of f_p_ variation was 62% and 93%, respectively.

**Conclusions:**

IVIM parameters represent a valuable tool in the evaluation of the anti-angiogenic therapy in patients with liver metastases from colorectal cancer. A percentage change of f_p_ represents the most effective DWI marker in the assessment of tumor response.

## Introduction

Colorectal cancer is the second most common cause of cancer deaths worldwide and approximately 50% of the patients will eventually develop distant metastases [1]. The treatment options for patients with advanced colorectal cancer have changed considerably over the past decades [1]. The standard chemotherapy scheme consists of a fluoropyrimidine, irinotecan, and oxaliplatin, which may be used either in combination or sequentially in the majority of patients [2, 3]. The prognosis for advanced colorectal cancer patients has been further improved by the use of a new class of targeted agents: bevacizumab, an antibody against the vascular endothelial growth factor [4], and cetuximab and panitumumab, antibodies against the epidermal growth factor receptor [4].

Despite the increased efficacy of treatment, only a subset of patients with metastatic colorectal cancer will respond. The availability of early predictive markers for response could therefore prevent unnecessary toxicity in non-responder patients and could also reduce the costs of treatment [5].

Diffusion-weighted imaging (DWI) supplies information of water proton mobility [6, 7]. This can be employed to assess the microstructural organization of a tissue like cell density, cell membrane integrity and ultimately cell viability which affects water diffusion properties in the extracellular space [7]. Le Bihan et al. illustrated the principles of intravoxel incoherent motion (IVIM) and suggested that using a more sophisticated approach to describe the relationship between signal attenuation in tissues and increasing b value would enable quantitative parameters that separately reflect tissue diffusivity and tissue microcapillary perfusion to be estimated [8]. IVIM data can be analyzed either quantitatively or qualitatively. Quantitative IVIM parameters may be useful for tissue characterization and assessment of tissue function, while qualitative analysis may be useful for the detection of pathology [9].

As a part of an ongoing study on the additional effect of the antiangiogenic drug bevacizumab in the neoadjuvant treatment of liver metastases we decided to include a functional MRI examination. Our purpose was to test if DWI could predict the tumor response in patients with colorectal liver metastases as early as two weeks after the bevacizumab-based therapy.

## Materials and Methods

### Patient Population

The study was approved by the review board of National Cancer Institute Pascale Foundation of Naples and written informed consent was obtained from each patient. From October 2011 to September 2013 we enrolled 22 patients (10 women and 12 men; mean age 52 years; range: 43–67 years) with unresectable liver metastases from a histologically proven previous or simultaneous colorectal carcinoma. Patients were managed by a combination of conventional chemotherapy and administration of an agent targeting the vascular endothelial growth factor (bevacizumab). The treatment rationale was to obtain an adequate reduction in liver metastasis to allow safe resection of residual liver disease. Patients were considered eligible according to the decision of our interdisciplinary tumor board and the unresectability status was defined by our surgeon. Patients with synchronous non-liver metastases were excluded. All patients in this series were monitorized with whole-body CT every 2 months. A subset of 12 non-consecutive patients (5 women and 7 men; mean age 55 years; range 45–67) were also investigated with functional MRI and these subjects formed our final study population. Ten patients were excluded by analysis because of contraindications to Magnetic Resonance examination (six patients) or absence of patient consensus for Magnetic Resonance examination (four patients). The DWI analysis was carried out on all metastases larger than one centimeter.

### Chemotherapy Protocol

Patients received neoadjuvant mFOLFOX6 (5- fluorouracil, leucovorin, oxaliplatin) plus bevacizumab. mFOLFOX6 was administered IV every 14 days with oxaliplatin 85 mg/m^-2^ by infusion on day 1, followed by leucovorin 200 mg/m^-2^ infusion on day 1, followed by 5-fluorouracil 400 mg/m^-2^ bolus on day 1, and 5-fluorouracil 2400 mgm^-2^ 46-h continuous infusion. The antiangiogenic drug Bevacizumab was administered every 14 days at 5 mg/kg by IV infusion over 90 minutes at the first cycle, and then, if adequately tolerated, over 60 minutes. The treatment of mFOLFOX6 plus bevacizumab was administered for 5 cycles, followed by one cycle of mFOLFOX6 without bevacizumab to prolong the bevacizumab-free interval and to reduce the risk of surgical bleeding.

### MR Imaging Protocol

Imaging studies were obtained at two time points, baseline (T0) at a mean of 5 days prior to treatment and 14 days after (T14), at the end of the first chemotherapy cycle.

MR Imaging was performed with a 1.5 T system (Magnetom Symphony, upgraded to Total Imaging Matrix Package, Siemens, Erlangen, Germany) employing body and surface coils. Imaging protocol included unenhanced, coronal, free-breathing true fast imaging with steady state free precession (TRUFI) T2-weighted scans; triggered-breathing half-Fourier acquisition single-shot turbo spin-echo (HASTE), axial T2-weighted scans with or without fat saturation (spectral adiabatic inversion recovery—SPAIR); end-expiratory breath-hold in-out of phase axial T1-weighted scans. An axial, free-breathing, single-shot transversal echo planar DWI was acquired using seven b value: 0, 50, 100, 150, 300, 600, and 800 s/mm^2^.

MRI protocols used are listed in Table 1.

**Table 1:** Pulse Sequence Parameters on MR studies.

Sequence	Orientation	TR/TE/FA (ms/ms/deg.)	TA (s)	Image size	Slice thickness / Gap (mm)	Base / phase resolution (ND/%)	Parallel imaging strategies
Trufi T2-W	Coronal	4.4/2.2/120	46	512x512	4 / 0	256/78	IPAT 2 GRAPPA
HASTE T2-W	Axial	1500/90/170	45	320x320	5 / 0	320/75	IPAT 2 GRAPPA
In-Out phase T1-W	Axial	160/2.35/70	33	256x192	5 / 0	256/90	IPAT 2 GRAPPA
DWI	Axial	7500/91/90	51	192x192	3 / 0	192/98	IPAT 2 GRAPPA

Note.–W = Weighted, TR = Repetition time, TE = Echo time, FA = Flip angle, TA = Acquisition time, ND = Non dimensional, IPAT = Integrated Parallel Acquisition Techniques, GRAPPA = generalized auto-calibrating partially parallel acquisitions, TRUFI = True fast imaging with steady state free precession, HASTE = Half-Fourier acquisition single-shot turbo spin-echo, DWI = Diffusion-weighted imaging.

### Images Analysis

In this study the diffusion parameters estimation was performed using a bi-exponential model describing the intravoxel incoherent motion [8, 9]. In fact, for a voxel with a large vascular fraction, the MRI data decay can deviate from a mono-exponential form, in particular showing a fast decay in the range of low b values generated by the IVIM effect [8, 9]. It is known that diffusion-weighted signal decay is commonly analyzed using the mono-exponential model (eq 1) for apparent diffusion coefficient (ADC):

$$\text{ADC}=\frac{\text{ln}\left(\frac{{\text{S}}_{0}}{{\text{S}}_{\text{b}}}\right)}{\text{b}}$$ (1)

Where S_b_ is the MRI signal intensity with diffusion weighting b, S_0_ is the non-diffusion-weighted signal intensity.

Thus, in addition to the mono-exponential model, we employed a bi-exponential model to estimate the IVIM-related parameters of pseudo-diffusivity (D_p_), perfusion fraction (f_p_), and tissue diffusivity (D_t_). This IVIM bi-exponential model is defined by (eq 2):

$$\frac{{\text{S}}_{0}}{{\text{S}}_{\text{b}}}={\text{f}}_{\text{p}\cdot }\text{exp}\left(-\text{b }\cdot {\text{D}}_{\text{p}}\right)+\left(1-{\text{f}}_{\text{p}}\right)\cdot \text{exp}\left(-\text{b}\cdot {\text{D}}_{\text{t}}\right)$$ (2)

We performed a segmented analysis procedure to estimate the three parameters because the bi-exponential model may often be ill-conditioned because of a limited number of samples, small perfusion fraction and/or similar compartmental diffusivities. Therefore, since D_p_ is typically significantly greater than D_t_ [2], when the b value is significantly greater than ~1/D_p_ (e.g. for D_p_~10 mm^2^/ms, b >100 s/mm^2^), the contribution of the pseudo-diffusion term to the signal decay becomes negligible. In this high b-value regime, the eq 2 can be simplified to a mono-exponential eq 1, whereby D_t_ and f_p_ can be estimated (eq 3):

$${\text{S}}_{\text{high}}={\text{S}}_{0}\left(1-{\text{f}}_{\text{p}}\right)\cdot \text{exp}\left(-\text{b}\cdot {\text{D}}_{\text{t}}\right)$$ (3)

Operationally, D_t_ is determined from a mono-exponential fit to data above a chosen threshold (b >200 s/mm^2^ in this study). With D_t_ determined using eq 3, D_p_, f_p_, and S_0_ values can be estimated using a nonlinear fit of eq 2 to the entire dataset that minimizes the residual sum of squares. In addition to the basic IVIM metrics, the product f_p_D_p_ (a quantity including both volume and velocity information) was considered as a parameter roughly analogous to flow as measured in perfusion imaging [8].

**Fig 1:**
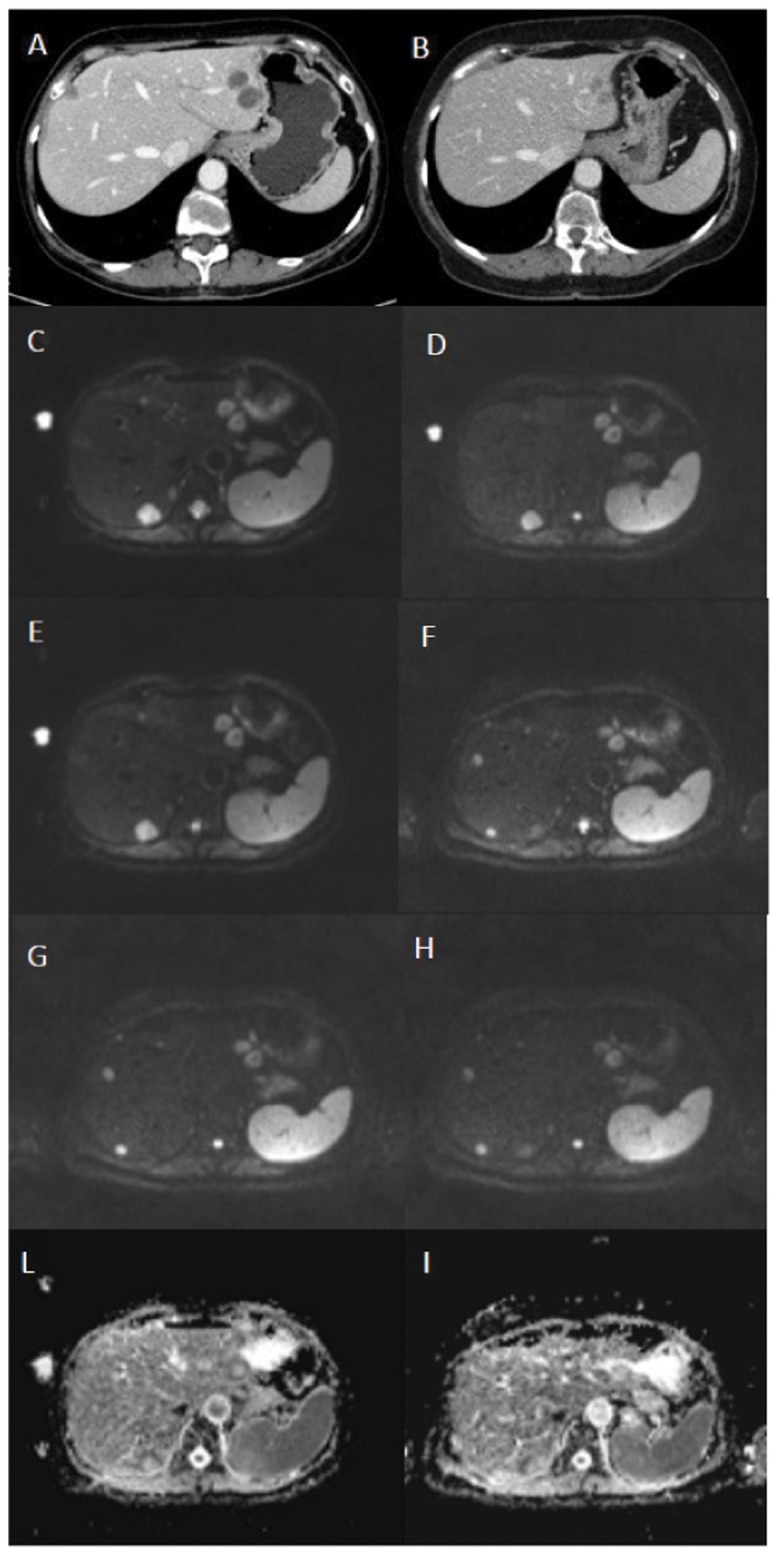
45-year-old woman with liver metastasis from colorectal cancer. Portal-phase axial CT scans before (A) and three months after (B) treatment show a decrease in size of two left liver lobe lesions and disappearance of the right liver lobe lesion. Axial DWI images before (C, b value 50, D, b value 400, E, b value 800) and 14 days after treatment (F, b value 50, G, b value 400, H, b value 800) do not demonstrate significant differences in the signal intensity of the two left liver lobe lesions with disappearance of the right liver lobe lesion. Axial tissue diffusion maps before (I) and 14 days after treatment (J) do not show significant differences in the signal intensity of the two left liver lobe lesions with disappearance of the right liver lobe lesion. This demonstrates that the qualitative assessment and the quantitative tissue diffusion-based assessment are not sufficient to evaluate tumor response.

This analysis was ROI-based using median value of single voxel signals for each b value in order to increase the signal noise ratio before perform diffusion curve fitting and to reduce error fitting (in terms of residual sum of squares). ROIs for the tumor lesions were manually drawn by expert radiologist, with 14 years of experience, including hyperintense voxels in comparison of background on diffusion weighted image at b value 800 s/mm^2^ (Fig 1I and 1J). No motion correction algorithm was used but ROIs were drawn taking care to exclude areas in which movement artifacts or blurring caused voxel misalignments.

The data analysis was performed using an in-house software written in Matlab (The MathWorks, Inc., Natick, USA).

### Statistical Analysis

The response to chemotherapy was evaluated according the RECIST [10, 11] as calculated on the portal-phase images of the routine whole-body CT scan obtained after three months from starting the treatment. The lesions that showed a decrease in the sum of longest diameters equal or greater than 30% in comparison with the baseline CT exam were considered respondent while the lesions showing a decrease in sum of longest diameters from baseline lower than 30% were considered not respondent (stable disease or progression disease).

Median diffusion parameters of ROI were used as representative values for each lesion (absolute value) and also the normalized diffusion parameters in comparison to median value of spleen were recorded [12]. Normalized values were obtained using the ratio between absolute value of diffusion parameter and the median value of same diffusion parameters in spleen.

The percentage change of the diffusion parameters between the time T0 and T14 were calculated. Mann Whitney test for unpaired data was used to individuate the statistical difference between the percentage change evaluated on absolute diffusion parameters and on normalized values. Wilcoxon test for paired data was used in order to individuate the statistical difference of the diffusion parameters between pre and post treatment. Mann Whitney test for unpaired data was used to individuate the statistical difference of diffusion parameters in respondent and non-responder lesion. Receiver operating characteristics (ROC) analysis was performed to identify the best diffusion parameter having larger area under the curve [13, 14] and to calculate for each diffusion parameter the optimal cut off value by means of Youden Index [15]. The sensitivity and specificity were reported in correspondence of optimal cut off values. A p value <0.05 was considered significant. Statistical analysis was obtained by means of the Statistic Toolbox of Matlab.

## Results

A total of 36 metastases were evaluated (1–5 per patient, mean 3). The size of the lesions before treatment ranged from 1 to 5 cm (mean, 3 cm). Twenty-four lesions were regarded as responders and twelve lesions as not responders, according to the RECIST (Fig 1).

**Fig 2:**
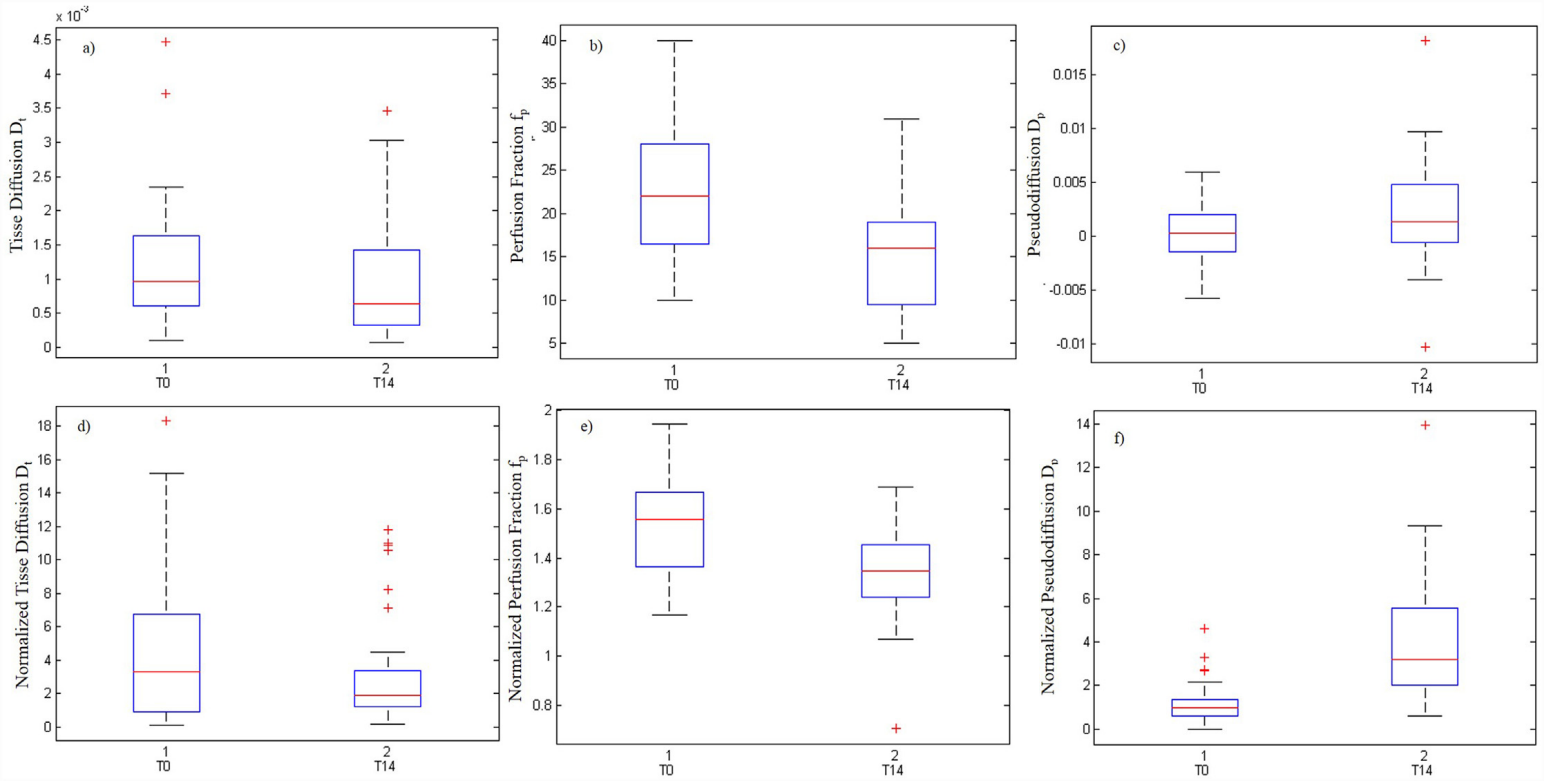
Boxplots of diffusion parameters in term of absolute (A, B, C) and normalized values (D, E, F). The middle line is the median value. The inferior and superior extremes of the box correspond to the first and third quartile respectively. The whiskers lines correspond to values within 1.5 times the interquartile range from the ends of the box. Outliner data beyond the ends of the whiskers are displayed with a + sign.

**Fig 3:**
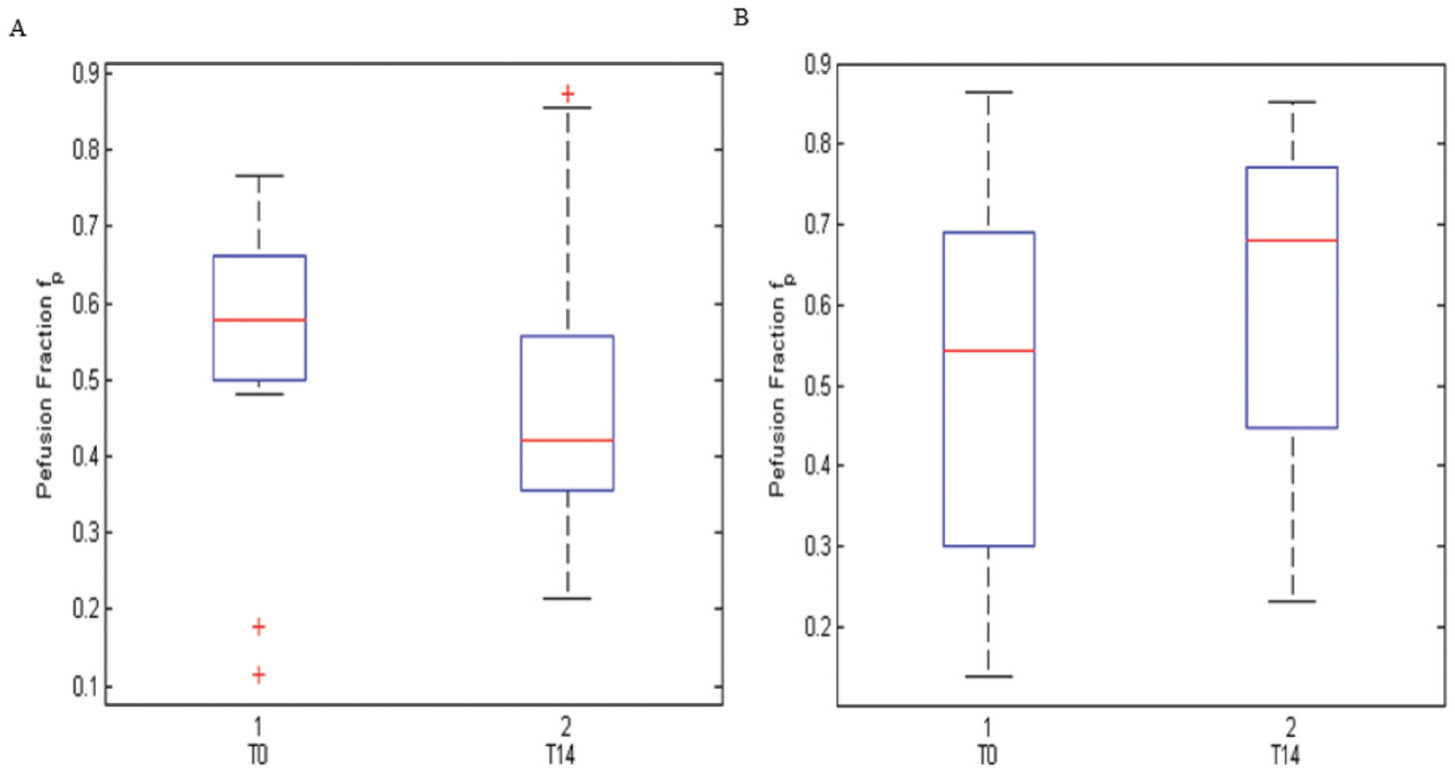
Boxplot of perfusion fraction f_p_. A, perfusion fraction values before and after treatment in responder patients. B, perfusion fraction values before and after treatment in non-responder patients. The middle line is the median value. The inferior and superior extremes of the box correspond to the first and third quartile respectively. The whiskers lines correspond to values within 1.5 times the interquartile range from the ends of the box. Outliner data beyond the ends of the whiskers are displayed with a + sign.

There was no statistically significant difference between the percentage change evaluated on absolute diffusion parameters and on normalized values (p value = 0.23, Mann Whitney Test). Fig 2 shows the changes of the absolute diffusion parameters and normalized values before and after therapy. Variation in the D_t_ and D_p_ was not an adequate index of the early changes of tumor microenvironment during chemotherapy in comparison of f_p_ variation and of the variation in the f_p_D_p_ product (p value = 0.01, Wilcoxon test) that were representative of early changes. Moreover, the percentage change values of perfusion fraction showed a statistical difference between responder by non-responder lesions at Mann Whitney Test (p value = 0.02). Fig 3 shows the boxplot of perfusion fraction f_p_, before and after treatment in responder patients (A) before and after treatment in non-responder patients (B).

**Fig 4:**
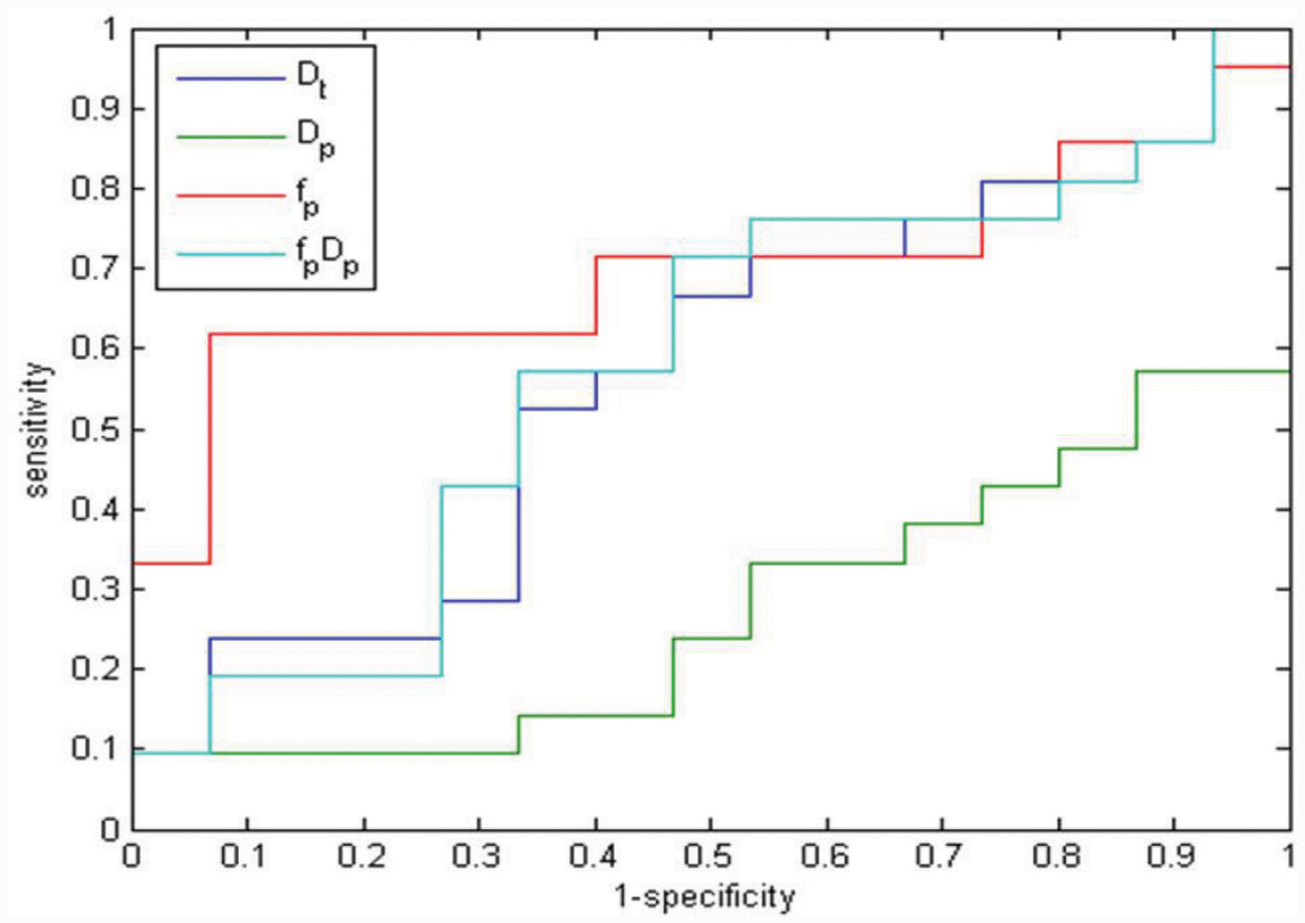
ROC analysis for D_t_, D_p_, f_p_, and f_p_D_p_ changes in discriminating responders from not responders: blue line correspond to ROC of D_t_ variation, green line correspond to ROC of D_p_ variation, red line correspond to ROC of f_p_ variation, and azure line correspond to ROC of f_p_D_p_ variation.

The f_p_ change obtained the largest Area Under Curve (AUC) in ROC analysis (Fig 4, Table 2). The f_p_ change sensitivity and specificity values were achieved compared to a cut-off equal to 54% (percentage change before and after treatment), and these were respectively of 62% and 93%.

**Table 2:** Sensitivity, specificity, accuracy, area under the curve (AUC), and cut-off value for changes in diffusion parameters between baseline and 14-day control.

	Sensitivity [%]	Specificity [%]	Accuracy [%]	AUC	Cut-off Value [%]
D_p_	67	53	61	0.57	24
D_t_	10	100	47	0.27	85
f_p_	62	93	76	0.70	54
f_p_ D_p_	71	53	64	0.51	15

## Discussion

To the best of our knowledge, this is the first study evaluating the sensitivity and specificity for the diffusion parameters in the early assessment of response to therapy (only after 14 days) in colorectal cancer metastasis. This may have a relevant practical value, allowing to determinate the effectiveness of therapy at the end of the first cycle of therapy and to change the therapy to improve the patient outcome and avoid at the same time an overtreatment.

Anatomical approaches based on measurements of tumor size [11, 12] have some limitations such as in the case of a lesion persisting after the therapy. So, more sophisticated measurement criteria and new imaging tools have been applied to the evaluation of treated lesions [16].

Functional imaging techniques are increasingly used to monitor response to therapies. DWI, that provides information on tissue cellularity, extracellular space tortuosity, and the integrity of cellular membranes by measuring the random motion of the water molecules in tissue, has been hypothesized as an effective tool to detect responder and non-responder lesions [17]. Since cellular death and vascular changes in response to treatment can both precede the changes in lesion size, DW-MRI may become an early biomarker for effective assessment of treatment response particularly for those therapies inducing apoptosis [17]. In particular, IVIM is a technique that enables the measurement of perfusion additional to diffusion. Consequently, DWI is the only imaging modality providing information about the lesions vascularization without any contrast medium injection [8, 9].

Most studies have shown that successful treatment is reflected by an increase in tumor ADC values. Rising ADC values with successful therapy have been noted in several anatomic sites, including breast cancers [18] and liver cancers [19]. In colorectal metastases Cui et al. [20] showed that the mean ADC of liver metastases responding to chemotherapy increases by 0.22 after one week of treatment, while nonresponding lesions do not change significantly. Koh et al. [21] measured liver metastases before and after chemotherapy and found that the responding lesions (measured a 150–500 b values) increased from a mean value of 1.15×10^−3^mm^2^/s before treatment to a mean value of 1.41×10^−3^mm^2^/s at the end of treatment. In our study there was no early rise of tissue diffusion or pseudodiffusion values. In our opinion this may depend on the short time interval from drug administration. The rationale for combined therapy of antiangiogenic plus cytotoxic agents is “normalizing tumor vasculature before its destruction” [22]. In fact tissue diffusion detects thermally-induced motion of water molecules and cellular integrity [7] and correlations between tissue diffusion and proliferation and apoptotic markers have been shown in an animal model before [23]. So we think that in such a short time interval there is only a perfusion effect and no apoptosis yet. Consequently, the tissue diffusion does no rise while f_p_ and the product f_p_D_p_, that reflect the vascular changes, show a modification being representative of the early perfusion effect of therapy.

Both D_p_ and f_p_ parameter derived from IVIM have been demonstrated to not correlate with the viable tumor or tumor necrosis on pathological analysis [24, 25]. In our study we have not tried to evaluate the tumor necrosis, since many lesions were already necrotic centrally before starting treatment. Instead, our focus was to early determine the effectiveness of bevacizumab basing on the vascularization changes. As a matter of facts, this drug, whose efficacy in the treatment of liver metastases has been established [26], is an antibody against vascular endothelial growth factor and so the difference in the f_p_ before and after therapy may reflect the changes of tumor microenvironment during chemotherapy.

Bevacizumab has several different mechanisms of action that result in inhibition of new vessel growth, regression of newly formed tumor vasculature, normalization of tumor blood flow, and direct effects on tumor cells. Lee et al., in a murine model, have shown that IVIM parameters, D_p_ and f_p_ values were significantly correlated with the microvessel density score and that DWI might be able to simultaneously provide information about tumor perfusion and diffusion [27]. Our results are in agreement with these experimental findings potentially providing for a diagnostic tool to be used in the clinical setting.

A recent study [28] concluded the measurement reproducibility of f_p_ and D_p_ estimates derived from the widely used non-linear least squares fitting is limited. Consequently, we estimated the parameters not only in absolute terms but also normalized to the spleen [10], without identifying any statistically significant differences between the two options.

Our study has a number of limitations. First, we had to refer to the RECIST system for comparison, although these criteria are notoriously limited in the early assessment of treatment response. However, RECIST represent by now the only standardized system available in the evaluation the therapeutic effect, despite their tendency to underestimate the treatment effectiveness. Some of our non-responders, particularly those with standard disease by RECIST are probably responders and this could have inevitably interfered with the final results. Secondly, we have to admit that the sample size of the lesions and, even more, of the patients in our series was small. By now we are enrolling new patients to boost the statistical significance and the clinical impact of our results. Additionally, we evaluated the patients only after the first cycle of chemotherapy, while after subsequent cycles the patients have been studied only with intravenous contrast-enhanced CT, so we do not have data on the changes of the parameters of DWI during the entire chemotherapy. Moreover, respiratory artifacts may have an impact on the images evaluation. However, no patient in our series was excluded because of the DWI images quality, also because motion misregistration has a minor impact on ROI-based analysis. At the end, a single expert radiologist with 14 year of experience performed a manual segmentation of lesions and inter-observer variability was not performed. Therefore, future goals are to estimate the reliability of diffusion parameters measurements including inter-observer variability and to assess whether and how these parameters change during the entire course of chemotherapy in order to provide biomarkers of response, to evaluate the feasibility and potential of DWI parameters to monitor surrounding healthy tissues during chemotherapy and to compare f_p_ and f_p_D_p_ with the semi-quantitative and quantitative data obtained with DCE-MRI.

## Conclusion

IVIM parameters represent a valuable tool in the evaluation of the effectiveness of anti-angiogenic therapy in patients with liver metastases from colorectal cancer. A percentage change of f_p_ discriminates with high specificity those lesions being responsive to treatment as early as at the end of the first cycle of chemotherapy. Consequently, the patients can benefit of a rapid change in the planned therapy when functional data fail to show a response.
